# On the Diseases of Infants and Children

**Published:** 1850-02

**Authors:** 


*On the Diseases of Infants and Children.* By FLEETWOOD CHURCHILL, M. D., M. R. I. A., etc. Philadelphia: Lea and Blanchard, 1850. 8 vo. pp. 636.

This is an original American publication, by a well known British writer. The circumstances under which it appears in this country, are stated by the author in the following paragraphs, quoted from the preface:

It is with much gratification that I acknowledge this volume to owe its existence to the solicitations of my excellent American publishers. After making a considerable collection of works on Diseases of Chileren, I had laid them aside, hopeless of accomplishing the task of writing the work I had contemplated; but it was impossible to decline an invitation so flattering, from a country which had shown so much indulgence to my former works.

I have, therefore, in such leisure as I have been able to command during the last three years, written this volume, not as the exponent of my own experience alone, but as embracing the information recorded by all the authors within my reach, of which I have freely availed myself, and if it prove useful and acceptable to my American brethren, I shall be richly repaid.

The time will soon arrive when foreign authors will be nothing loath to follow the example of Dr. Churchill. If we are correctly informed, the medical public in this country, in the aggregate, are far better patrons of medical literature than in Europe. With the increasing magnitude of the profession, then, it is altogether probable that ere long, if not now, British writers will be anxious to reap the fruits of a transatlantic currency. We doubt not the liberal publishers of this work were justified in offering an adequate inducement for ite preparation. And with this there can be no ground for dissatisfaction, provided domestic productions of equal excellence are not depreciated by an undue preference for an imported article. Were this the case, the fault would be with the medical profession, not with the publishers.

Dr. Churchill's previous publications will be regarded as affording a sufficient guaranty for the value of the present work. In so far as we may judge from a partial examination, it fully sustains the author's reputation as a candid, judicious writer. We are pleased to see that he has not neglected proper reference to the several valuable treatises which have, within the past few years, emanated from American authors.

